# Neutropenic enterocolitis-induced sepsis and disseminated intravascular coagulation after chemotherapy: a case report

**DOI:** 10.1186/s12905-021-01302-8

**Published:** 2021-05-03

**Authors:** Masako Ishikawa, Kentaro Nakayama, Sultana Razia, Akiko Ishida, Hitomi Yamashita, Tomoka Ishibashi, Seiya Sato, Kiyoka Sawada, Hiroki Sasamori, Sonomi Kurose, Noriyoshi Ishikawa, Satoru Kyo

**Affiliations:** 1 Department of Obstetrics and Gynecology, Shimane University Faculty of Medicine, Enyacho 89-1, Izumo, Shimane 6938501 Japan; 2 Shimane University Hospital Postgraduate Clinical Training Center, Izumo, Shimane Japan; 3 Department of Organ Pathology, Shimane University Faculty of Medicine, Izumo, Shimane Japan

**Keywords:** Endometrial cancer, Neutropenic enterocolitis, Septic shock, Disseminated intravascular coagulation, Ileus, Bacterial translocation, Obesity paradox

## Abstract

**Background:**

Neutropenic enterocolitis (NE) is a potentially life-threatening disease that primarily occurs in cancer patients treated with chemotherapy. NE has substantial morbidity and mortality, and its incidence has increased with the widespread use of chemotherapeutic agents such as taxanes, gemcitabine, and leucovorin in patients with lung, breast, gastric, and ovarian cancers. Sometimes NE can be a possible cause of death. Although, conservative approaches are often successful, there are currently no standardized treatment guidelines for NE and it is unclear when such strategies should be implemented. Therefore, we present this report to provide a greater insight into the possible treatment of NE.

**Case presentation:**

We report the case of a 72-year-old woman with endometrial cancer who was undergoing treatment for hypertension, obesity and diabetes mellitus. The patient initially developed paralytic ileus on the 6th postoperative day (POD) after surgery for endometrial serous carcinoma. Complete recovery was achieved after 4 days of fasting and fluid replacement therapy. On the 27th POD, she received the first cycle of combination chemotherapy consisting of paclitaxel and carboplatin. On day 5 of chemotherapy, she developed the systemic inflammatory response syndrome including febrile neutropenia and sepsis. She then developed disseminated intravascular coagulation (DIC) and septic shock. The patient was subsequently moved to the intensive care unit (ICU). Despite initiating the standard treatment for septic shock and DIC, her overall status worsened. It was assumed that gut distention had led to bowel damage, subsequently leading to bacterial translocation. Thus, she developed NE with severe DIC and septic shock. We decided to reduce the intestinal pressure using an ileus tube to suction the additional air and fluid, even though doing so had a risk of worsening her general condition. The inflammatory reaction subsided, and her general condition improved. The patient recovered after 18 days in the ICU and was discharged alive.

**Conclusions:**

Herein, we describe a patient with suspected chemotherapy-associated NE. Our observations suggest that postoperative ileus may be one of the possible causes of NE. Patients who experience postoperative ileus must be carefully monitored while undergoing chemotherapy.

## Back ground

The incidence of endometrial carcinoma has been increasing recently [1], including in Japan [2]. Patients with endometrial carcinoma generally have a good prognosis owing to the significant advances in diagnostic and therapeutic modalities [3]. Chemotherapy is the mainstay treatment of endometrial carcinoma. However, while patients rarely experience life-threatening adverse events, some chemotherapeutic drugs have been found to be associated with gastrointestinal emergencies such as neutropenic enterocolitis (NE) (Table 1) [4–18]. NE is an acute life-threatening condition that is characterized by transmural inflammation of the cecum. It often involves the ascending colon and ileum, and can sometimes lead to severe patient outcomes [19, 20].

**Table 1:** Previous reports of gastrointestinal emergencies induced by Anti cancer drugs in patients with malignant disease

Disease	Age (year)/sex	Chemotherapy regimen	Dead/alive	References
Lung cancer	68/F	Paclitaxel 210 mg/m^2^, Carboplatin (AUC 6)	Alive	[4]
	70/M	Docetaxel 100 mg/m^2^	Recovered	[5]
	68/M	Docetaxel 100 mg/m^2^, Cisplatin 80 mg/m^2^	Sigmoiditis	
	72/F	Docetaxel 75 mg/m^2^, Flavopiridol 60 mg/m^2^	Death	[6]
	18–75	Docetaxel 75 mg/m^2^, Cisplatin 75 mg/m^2^		[7]
	72/M	Docetaxel (35 mg/m^2^)	Death	[8]
	46/M	Docetaxel 100 mg/m^2^	Death	[9]
Breast cancer	53/F	Docetaxel 60 mg/m^2^, Vinorelbine 20 mg/m^2^	Recovered	[10]
	51/F	Docetaxel 75 mg/m^2^, Vinorelbine 20 mg/m^2^	Death	
	68/F	Docetaxel 75 mg/m^2^, Vinorelbine 20 mg/m^2^	Death	
	62/F	Docetaxel 75 mg/m^2^	Recovered	
	52/F	Docetaxel 90 mg/m^2^, Pamidronate 90 mg	Recovered	
	51/F	Docetaxel 60 mg/m^2^, Cyclophosphamide 450 mg/m^2^	Recovered	
	35/F	Paclitaxel, 180 mg/m^2^, Doxorubicin 75 mg/m^2^	Recovered	[11]
	46/F	Paclitaxel, 180 mg/m^2^, Doxorubicin 75 mg/m^2^	Recovered	
	57/F	Epirubicin 70 mg/m^2^, Docetaxel 80 mg/m^2^	Recovered	[5]
	72/F	Docetaxel 100 mg/m^2^, Mitoxantrone 8 mg/m^2^	Recovered	
	66/F	4 cycle of Taxotere and Cyclophosphamide	Recovered	[12]
	81/F	Docetaxel weekly (35 mg/m^2^)	Death	[8]
	61/F	Doxetaxele 100 mg/m^2^, doxorubicin 50 mg/m^2^, Cyclophosphamide 600 mg/m^2^ (after third cycle)	Recovered	[13]
Ovarian cancer	60/F	Paclitaxel 175 mg/m^2^, Carboplatin 6 mg/ml/min	Recovered	[14]
	62/F	Paclitaxel 175 mg/m^2^, Carboplatin (AUC 5), Gemcitabine 800 mg/m^2^ (after 1st cycle)	Death	[15]
	53/F	Paclitaxel (no dose written)	Recovered	[16]
	40/F	Paclitaxel (no dose written)	Recovered	
	63/F	Paclitaxel (no dose written)	Death	
	54/F	Paclitaxel (no dose written)	Recovered	
	43/F	Paclitaxel (no dose written)	Recovered	
	58/F	Paclitaxel (no dose written)	Death	
	70/F	Paclitaxel (no dose written)	Death	
	41/F	Paclitaxel 135 mg/m^2^, Cisplatin75 mg/m^2^	Death	[17]
	80/F	Paclitaxel 135 mg/m^2^	Death	
	47/F	Paclitaxel 135 mg/m^2^	Recovered	
	66/F	Paclitaxel 200 mg/m^2^, Cisplatin 70 mg/m^2^, Ifosfamide 1.5 g/m2	Recovered	[5]
Oesophageal cancer	Unknown	Paclitaxel 80–110 mg/m^2^, Cisplatin 70 mg/m^2^	Death (2 out of 4)	[18]
Endometrial cancer	72	Paclitaxel 175 mg/m^2^, Carboplatin AUC5	Recovered	Current case

Previous studies on the pathogenesis of NE indicate that the main factors involved in disease onset appear to be an intestinal mucosal injury together with the immunocompromised state and neutropenia of the impaired patients. These beginning conditions lead to a disrupted mucosal surface, engorged vessels, and intestinal edema, which becomes more vulnerable to bacterial intramural invasion [21]. Stemmler et al. [8] also reported that bacterial invasion, mucosal damage of the bowel, increased rapid growth of bacteria resulting from decreased immunocompetence, production of intramural hemorrhage, ulceration, bacterial endotoxins, ischemia, and in some cases, necrosis of the bowel wall and perforation.

NE has also been reported after treatment with vinorelbine [10, 22], gemcitabine [15], and especially taxanes [4–20, 22, 23] in patients with lung, breast, gastric, ovarian, or peritoneal malignancies and in patients with long-term neutropenia [4–8, 12–15, 24, 25]. According to previous reports, carboplatin is also more likely to cause neutropenia; however, it has specifically never been mentioned as the cause of NE, because its role in its pathogenesis remains incompletely understood and it is often used in combination with taxanes [4–10, 14, 15]. Docetaxel may result in an inflammatory bowel syndrome that clinically mimics pseudomembranous colitis [26]. Gastrointestinal mucosal toxicity and ileus have been described as some of its adverse effects [27]. While some information on NE is available in literature, it is very difficult to predict the factors that lead to NE in each patient.

Herein, we describe a novel case of uterine endometrial cancer that led to critical NE complicated by disseminated intravascular coagulation (DIC) and septic shock.

## Case presentation

**Fig. 1:**
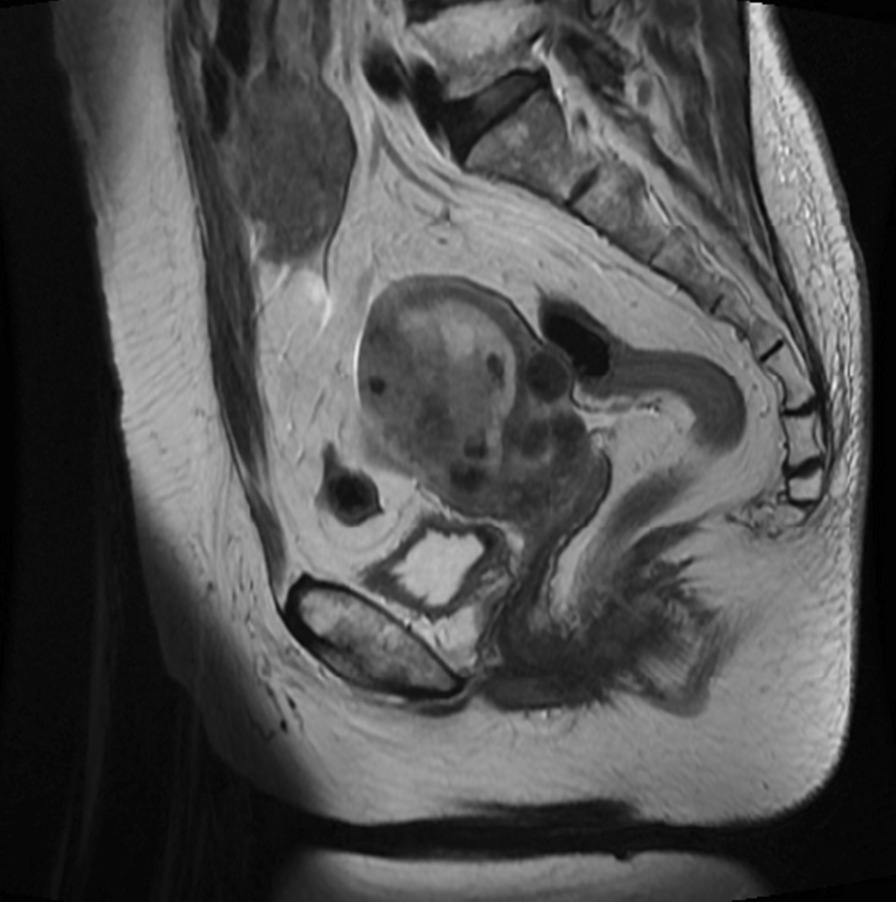
Magnetic resonance imaging of the pelvis. T2-weighted imaging reveals a thickened endometrial mass (60 × 66 × 53 mm)

**Fig. 2:**
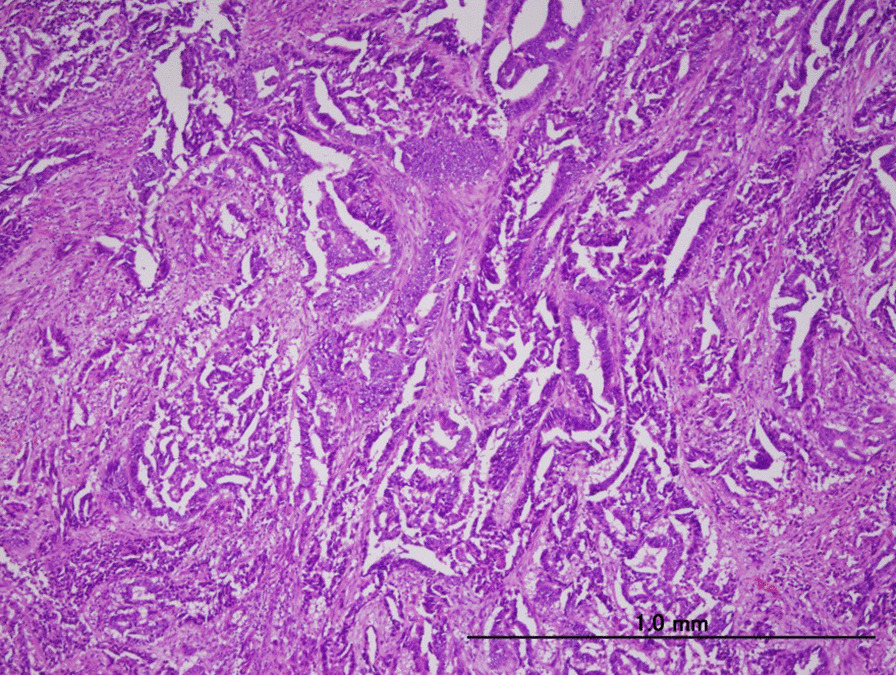
Pathological diagnosis: serous carcinoma

A 72-year-old Japanese woman on medications for hypertension, obesity, and diabetes mellitus, was referred to our hospital with abnormal uterine bleeding. She was previously diagnosed with abnormal endometrial cytology at a previous clinic and visited our hospital for further examination and treatment. Endometrial biopsy revealed an adenocarcinoma (G2), while pelvic magnetic resonance imaging revealed a lesion mass of size 60 × 66 × 53 mm in the right side of the uterus (Fig. 1), which was suspected to invade the serosal side. An enlarged lymph node measuring 18 mm was also found in the lymph node clusters in the right internal iliac artery and the obturator lymph node. Therefore, hysterectomy, bilateral adnexectomy, pelvic lymph node dissection, and para-aortic lymph node dissection were performed. A pathological examination using surgical specimen revealed endometrial serous carcinoma (Fig. 2). Finally, she was diagnosed with stage III C2 endometrial cancer.

**Fig. 3:**
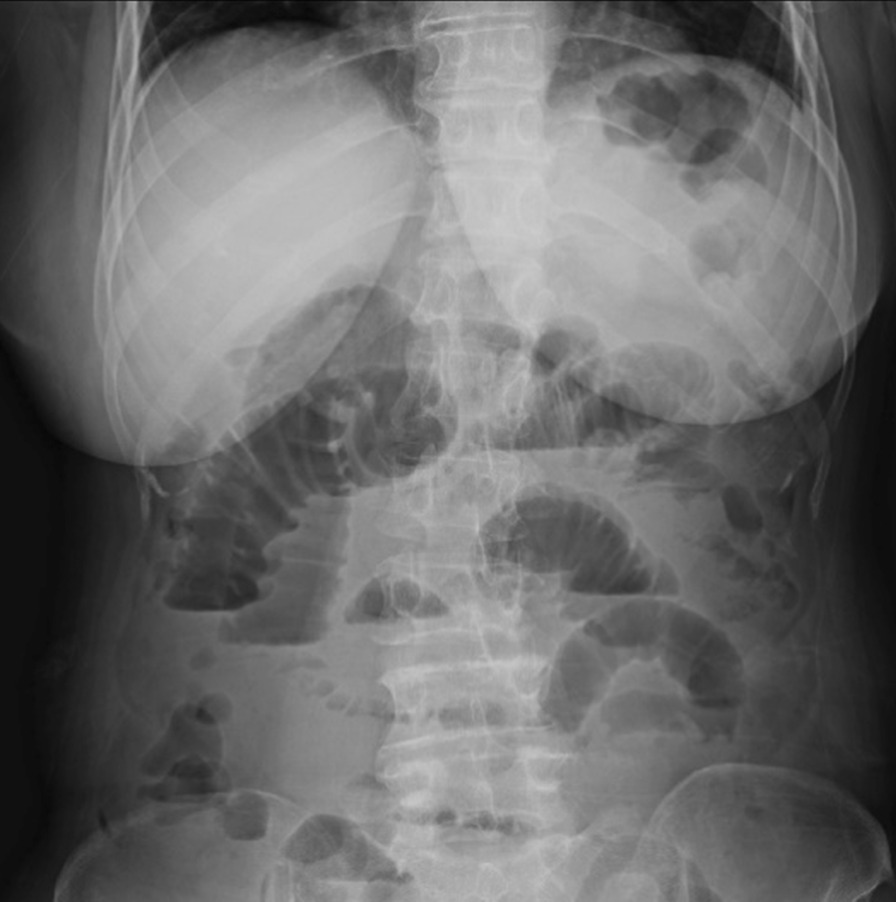
Abdominal X-ray demonstrating niveau formation in 6th POD

On the 6th postoperative day (POD), the patient developed abdominal bloating and nausea. Abdominal radiography revealed that the stomach and the intestine were markedly dilated with gas and air-fluid levels, indicating paralytic ileus (Fig. 3). Thus, the patient was initiated on fasting and fluid replacement therapy, following which she recovered completely within 4 days. On the 27th POD, the patient received the first cycle of combination chemotherapy consisting of paclitaxel (175 mg/m^2^; 3-h infusion) and carboplatin (at a dose corresponding to an area under the curve [AUC] of 5 mg/mL/min).

On day 5 of chemotherapy, the patient developed a fever (38.6 °C), diarrhea, and fatigue. Her pulse rate, respiratory rate, and blood pressure were 120 beats/min, 20 breaths/min, and 88/52 mmHg, respectively. Blood examination revealed a white blood cell count, neutrophil count, and C-reactive protein (CRP) level of 270/μL, 40/μL, and 17.92 mg/dL, respectively, which were suggestive of the systemic inflammatory response syndrome including febrile neutropenia (FN) and sepsis. Therefore, an antibiotic therapy and granulocyte-colony stimulating factor therapy were initiated immediately. However, her condition worsened the next day. The body temperature, pulse rate, respiratory rate, blood pressure, white blood cell count, neutrophil count, and CRP level were 40.2 °C, 150 beats/min, 49 breaths/min, 63/42 mmHg, 150/μL, 0/μL, and 41.5 mg/dL, respectively.

**Fig. 4:**
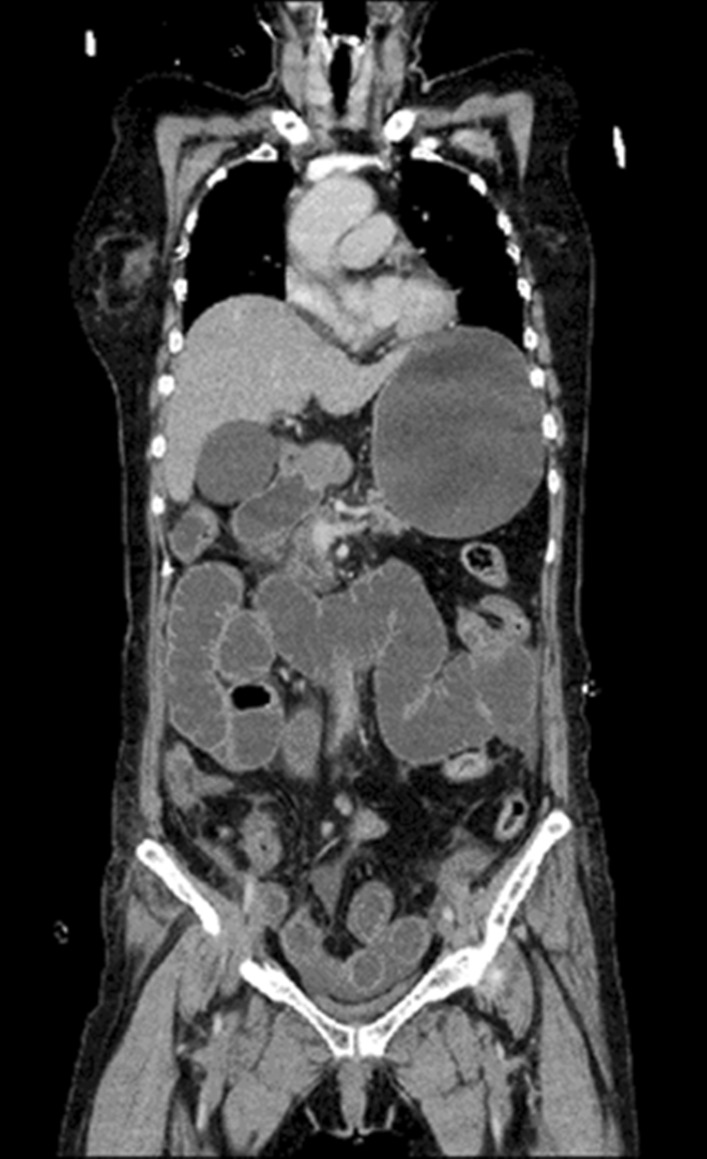
CT scan demonstrating remarkable intestinal dilation and thickening of the intestinal wall on the 5th day after the first chemotherapeutic cycle

**Fig. 5:**
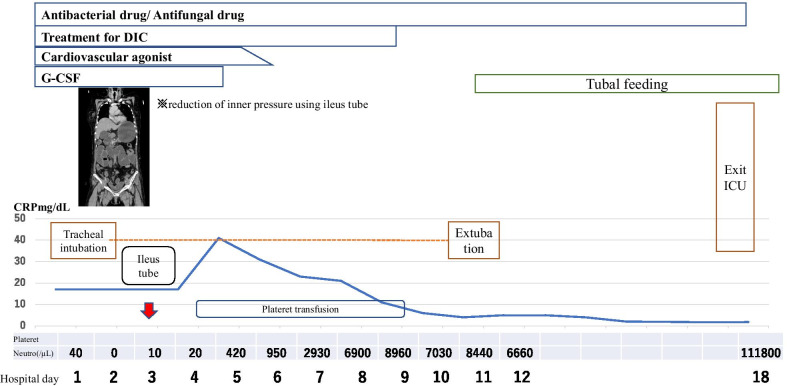
Developments after hospital admission. This chart demonstrates the progress of the patient’s condition

Because she suffered from septic shock and DIC, she was shifted to the intensive care unit (ICU). Computed tomography (CT) of the abdomen and pelvis revealed remarkable intestinal dilation and thickening of the intestinal wall. Therefore, we considered enteritis as the origin of inflammation (Fig. 4). Furthermore, we speculated that the patient’s condition was associated with toxicity due to bacterial translocation. However, no bacteria were detected in the blood and intestinal fluid. In addition to the treatment for septic shock and DIC, a long ileus tube was inserted into the stomach through the nasal cavity because she experienced a constant high-pressure feeling in the intestine and because we had to suction the extra air and fluid. Favorable outcomes were achieved, including reduced edema in the intestinal colon, improved circulation in the involved intestine, and correction of the intestinal kinking. Her condition gradually improved, and the neutrophil count and immune function improved from the 4th day of ICU admission. She was discharged alive and well from the ICU after 18 days (Fig. 5). The patient’s clinical course is shown in Fig. 5. Written informed consent was obtained from the patient for participating in all procedures, and this work was approved by the Institutional Review Board of the Shimane University (IRB No-20200110-1).

## Discussion and conclusions

The true incidence of NE is unknown [27]. While NE was initially reported after the use of taxane drugs, recent reports have highlighted the occurrence of NE after the administration of several chemotherapeutic drugs such as gemcitabine, leucovorin, cytosine arabinoside, vincristine, doxorubicin, cyclophosphamide, and 5-fluorouracil [20]. Furthermore, treatment with immunosuppressants (for organ transplantation), antibiotics, and sulfasalazine (for rheumatoid arthritis) has also been reported to cause NE [28, 29]. In the present case, the patient developed NE with septic shock and DIC after receiving the first cycle of adjuvant chemotherapy with platinum and a paclitaxel-based regimen for an endometrial serous carcinoma.

NE easily occurs in patients with neutrophil counts < 500/μL. Cardona et al. [24] reported that patients with neutrophil counts < 1000/μL had a higher risk of NE than those with counts > 1000/μL. Moreover, Wade et al. [25] reported that leukocyte count recovery after the onset of NE is associated with survival. Sharma et al. [30] found that 12% of the patients with epithelial ovarian cancer developed neutropenic fever while receiving first-line adjuvant chemotherapy. Markman et al. [31] also reported that among patients who underwent a carboplatin-based regimen for pelvic malignancies, FN occurred in 1% of those treated with carboplatin and paclitaxel and in 12% of those treated with carboplatin and docetaxel.

Herein, we present the case of a patient who was critically ill due to NE. To our knowledge, this is the first report on sepsis and DIC resulting from NE due to ileus following treatment for endometrial cancer. While there are a few reports on NE, it is necessary to recognize the considerable increase in the number of patients who are likely to experience an ileus.

Until now, intestinal mucosal damage from chemotherapeutic drugs has been considered as the cause of NE. However, the current case presents postoperative ileus as another possible cause of NE. Because ileus induces the elevation of the internal pressure in the intestine, it may lead to bacterial translocation, which may then progress to NE. This patient first developed paralytic ileus on the 6th POD and subsequently developed septic shock and DIC on the 6th day after the first chemotherapeutic cycle. Because she was at a high risk for ileus development, we managed the bowel peristalsis. Furthermore, we were also required to closely manage the NE, because she had received a taxane regimen. A similar case of a colon cancer patient who developed NE during postoperative chemotherapy has been reported [32]. Physicians should carefully consider chemotherapy in patients who develop paralytic ileus after surgery. In our case, the patient received paclitaxel, which is a known causative factor of NE. In similar cases, physicians should also consider the administration of medications at the beginning of chemotherapy for preventing paralysis of the intestine.

The outcome of this case also indicates the importance of simultaneous treatments for septic shock, DIC, and NE. For patients who initially develop NE, followed by DIC and septic shock, treatment should be initiated for not only DIC and septic shock, but also for the cause of septic shock. In such patients, septic shock would generally occur due to bacterial translocation triggered by an increase in the intra-intestinal pressure. Thus, the intra-intestinal pressure must be reduced primarily by using an ileus tube for aspirating the fluid and gas. In the current case, it was the only treatment deemed feasible for relieving the pressure, although it was challenging to insert the ileus tube due to the patient’s severely ill condition. Favorable outcomes were achieved, and the patient recovered. Her condition was managed by suctioning the extra air and fluid using an ileus tube along with the administration of broad spectrum antibiotics. This shows that the insertion of an ileus tube is essential for the curative treatment in critically ill patients, such as in this case. In the present case, we encountered severe NE after the first chemotherapeutic cycle with taxanes and platinum, which are routinely used for the treatment of patients with endometrial cancer in our institute. This is only a case report; therefore, it should not be assumed that every patient would survive a similar condition. However, it is very important for physicians who successfully treat similar cases having life-or-death situations to submit reports on the same.

Another notable point is that the patient was clinically obese. Her body mass index (BMI) was 30.4 kg/m^2^. The obesity paradox has been reported in various diseases such as cardiac diseases, type 2 diabetes mellitus, renal diseases, and cancers [33–35]. Some studies have reported the association between the obesity paradox and septic shock not caused by NE [36–38]. However, the evidence on the association between obesity and mortality is conflicting. For example, Oliveros et al. [39] and Wurziger et al. [40] demonstrated that obesity influences the risk for mortality. In contrast, Hogue et al. [41] concluded that the BMI does not affect mortality in critically ill patients. According to previous reports, a greater number of obese patients survive severe conditions in the ICU as compared to patients having a normal weight [38]. The observations in the present case were consistent with those of these previous reports. However, this case had certain limitations in that it was a single event and the factor whose addressal would contribute the most to the resolution of the condition was unknown.

From this case we learnt that the major and popular treatments involving the combination of taxanes and platinum for endometrial cancer patients can induce NE, which can also lead to sepsis and DIC after bacterial translocation. We yearn for residents to keep this in consideration. Postoperative ileus can cause clinical conditions similar to in this case, because the ischemic intestinal tract can easily undergo ileus again and experience bacterial translocation following an increase in the inner intestinal pressure. It is vital to ensure that these points are considered at the beginning of the first chemotherapeutic cycle.

In conclusion, postoperative ileus might be a risk factor of NE. Patients who experience ileus should be carefully evaluated for concurrent neutropenia when they receive chemotherapy. In case of NE with intestinal distention, it is essential to alleviate the inner pressure of the intestine to help remove the cause of bacterial translocation immediately.

## Data Availability

The datasets used and/or analysed during the current study available from the corresponding author on reasonable request.

## Abbreviations

NE: Neutropenic enterocolitis
DIC: Disseminated intravascular coagulation
G-CSF: Granulocyte-colony stimulating factor
SIRS: Systemic inflammatory response syndrome
ICU: Intensive care unit
BMI: Body mass index
MRI: Magnetic resonance imaging
AUC: Area under the curve
FN: Febrile neutropenia
CT: Computed tomography
